# Principles and Practice of Dental Surgery

**Published:** 1852-10

**Authors:** 


*Principles and Practice of Dental Surgery.*—Messrs. Lindsay & Blakiston, the publishers, have issued a fifth edition of the above named work. The improvements introduced in the present edition may be inferred from the following quotation from the preface:

"The whole work has been revised, and numerous additions made to almost every chapter. In the part devoted to mechanical dentistry three new chapters have been added. The first, treats on the composition, manufacture and methods of mounting block teeth; the second, on single teeth with a continuous artificial gum, uniting the teeth to each other and to the plate on which they are mounted; and the third, on the use of tin as a base for dental substitutes for the lower jaw. Thirty-five new engravings have also been added."

